# Novel Functional Changes during Podocyte Differentiation: Increase of Oxidative Resistance and H-Ferritin Expression

**DOI:** 10.1155/2014/976394

**Published:** 2014-07-06

**Authors:** Emese Bányai, Enikő Balogh, Miklós Fagyas, Paolo Arosio, Zoltán Hendrik, Gábor Király, Gábor Nagy, Bence Tánczos, István Pócsi, György Balla, József Balla, Gáspár Bánfalvi, Viktória Jeney

**Affiliations:** ^1^Department of Medicine, University of Debrecen, Debrecen 4032, Hungary; ^2^Division of Clinical Physiology, Institute of Cardiology, University of Debrecen, Debrecen 4032, Hungary; ^3^Department of Molecular and Translational Medicine, University of Brescia, 25125 Brescia, Italy; ^4^MTA-DE Vascular Biology, Thrombosis and Hemostasis Research Group, Hungarian Academy of Sciences, Debrecen 4032, Hungary; ^5^Department of Microbial Biotechnology and Cell Biology, University of Debrecen, Debrecen 4010, Hungary; ^6^Department of Pediatrics, University of Debrecen, Debrecen 4032, Hungary

## Abstract

Podocytes are highly specialized, arborized epithelial cells covering the outer surface of the glomerular tuft in the kidney. Terminally differentiated podocytes are unable to go through cell division and hereby they are lacking a key property for regeneration after a toxic injury. Podocytes are long-lived cells but, to date, little is known about the mechanisms that support their stress resistance. Our aim was to investigate whether the well-known morphological changes during podocyte differentiation are accompanied by changes in oxidative resistance in a manner that could support their long-term survival. We used a conditionally immortalized human podocyte cell line to study the morphological and functional changes during differentiation. We followed the differentiation process for 14 days by time-lapse microscopy. During this period nondifferentiated podocytes gradually transformed into large, nonproliferating, frequently multinucleated cells, with enlarged nuclei and opened chromatin structure. We observed that differentiated podocytes were highly resistant to oxidants such as H_2_O_2_ and heme when applied separately or in combination, whereas undifferentiated cells were prone to such challenges. Elevated oxidative resistance of differentiated podocytes was associated with increased activities of antioxidant enzymes and H-ferritin expression. Immunohistochemical analysis of normal human kidney specimens revealed that podocytes highly express H-ferritin *in vivo* as well.

## 1. Introduction

During the glomerular morphogenesis, four resident cell types are responsible for the formation of a functional glomerular filtration barrier in the kidney. A complex system of interference between parietal epithelial cells of Bowman's capsule, podocytes (visceral epithelial cells), fenestrated endothelial cells, and mesangial cells maintains the integrity of the glomerular structure. The parietal epithelial cells and the podocytes originate from the metanephric mesenchyme and are identifiable at week 5 of gestation in humans [1].

During embryonic development, the ability of podocytes to proliferate ceases, and the cells undergo cytoskeletal rearrangement, form foot processes, and express molecules that contribute to the formation of the slit diaphragm. Mature podocytes are highly specialized cells with an interdigitating network of foot processes which control the filtration of proteins from the capillary lumen into Bowman's space [2]. Narrow slits between the pedicles allow small molecules to pass while constituting a barrier against the filtration of large proteins. It is now widely accepted that podocytes are primarily responsible for the prevention of proteinuria. Under physiological conditions, terminal podocyte differentiation is accompanied by irreversible growth arrest, resulting in a limited lifetime of these cells [3]. In response to different kinds of injuries, podocytes go through cytological changes, like cell hypertrophy and foot processes retraction [4], which terminate in podocyte detachment and cell loss.

The glomerular filtration barrier is the primary site of sieving and is exposed to an excessive amount of circulating reactive oxygen species (ROS) [5]. Accumulating evidence suggests that oxidative stress is an important factor contributing to podocyte loss and eventually proteinuria [6–8]. Little is known about the presence and nature of defence mechanisms that could support long-term survival of differentiated podocytes in this challenging environment.

Antioxidant enzymes including glutathione-peroxidase (GPX), catalase, and superoxide dismutase (SOD) are crucial elements of the cellular defence system against ROS [9]. Damaging effect of ROS is highly exacerbated in the presence of transition metals, such as iron via a Fenton-reaction mediated way. Ferritin (450 kD) is an iron sequestering protein with the ability of iron detoxification and iron reserve. It has a highly conserved structure with 24 protein subunits composed of heavy chains (FtH) and light chains (FtL) in human. Heavy chains possess ferroxidase activity required for the incorporation of iron into the core [10]. Expression of the FtH and FtL subunits is regulated by iron at a posttranscriptional level and by oxidative stress at a transcriptional level [11–13].

The antioxidant nature of ferritin was proved* in vitro* [14] and more recently* in vivo* in conditional FtH knockout animals [15]. This mouse model provided evidence that the iron storage function of ferritin plays a major role in preventing the accumulation of free iron and reactive oxygen species in cells, thereby impeding organ damage [15, 16].

Our aim was to explore novel functional changes during podocyte differentiation that could support oxidative resistance and long-term survival of these cells. We hypothesized that antioxidant enzymes and FtH are an integral part of podocyte defence mechanism. In this work we applied time-lapse video microscopy to follow morphological changes of individual podocytes in the course of differentiation under* in vitro* growth conditions using a conditionally immortalized human podocyte cell line [17]. We showed that differentiated podocytes are more resistant to oxidative stress than nondifferentiated cells. Uncovering the background of elevated oxidative resistance, we found that activities of antioxidant enzymes GPX, catalase, and SOD are increased in differentiated podocytes compared to nondifferentiated cells. Moreover, we identified the cytoprotective effect of FtH that is robustly expressed in mature podocytes* in vitro* and* in vivo.* This paper provides evidence that the differentiation of immortalized human podocytes is a complex process showing characteristic phenotypic plasticity. Phenotypic changes during podocyte differentiation are not just a certain filtering function of podocytes but are of crucial importance in helping survival of these highly differentiated cells in certain oxidative conditions.

## 2. Materials and Methods

### 2.1. Cell Line and Growth Conditions

Human podocytes were kindly provided by Moin Saleem (University of Bristol) and cultured as described previously [17]. Briefly, cells were propagated at 33°C under permissive condition in RPMI containing 10% fetal bovine serum, antibiotics (10,000 units/mL penicillin G, 10 mg/mL streptomycin, and 25 *μ*g/mL amphotericin B), insulin (10 *μ*g/mL), transferrin (5.5 *μ*g/mL), and selenium (5 ng/mL Na selenite). At ~50–60% confluence, the cells were transferred to 37°C (nonpermissive condition) for 14 days to silence the temperature sensitive tsA58 T antigen. Passage numbers were used between 14 and 20. Cell culture materials were obtained from PAA Laboratories Ltd. (Cölbe, Germany).

### 2.2. Immunofluorescent Staining of Podocytes

Human immortalized podocytes were grown on poly-D-lysine-four-well chamber slides to 50–60% confluence. After removing the medium, cells were washed twice thoroughly with PBS followed by fixation with 4% freshly prepared paraformaldehyde and blocking with 5% donkey serum for 20 minutes. The cells were stained with primary anti-synaptopodin (sc-21537, 1 : 200, Santa Cruz Biotechnology Inc., Heidelberg, Germany) and secondary Cy3-conjugated antibodies. For the visualization of the cytoskeleton, fluorescein isothiocyanate- (FITC-) labelled phalloidin (Sigma-Aldrich, St. Louis, MO, USA) at a dose of 50 *μ*g/mL for 40 minutes was applied to stain F-actin. To optimize detection, streptavidin/biotinylated horse-radish peroxidase system was applied for amplification of the signal. Finally, the samples were covered with a DAPI (4′,6-diamidino-2-phenylindole) containing antifade mounting medium (EverBrite mounting medium, Biotium Inc., Hayward, CA, USA) to stain the nuclei and minimize photobleaching of the samples. Pictures were taken using a Leica DM4000 fluorescent microscope equipped with a Leica DFC310 FX digital color camera (Leica Microsystems GmbH, Wetzlar, Germany).

### 2.3. Time-Lapse Videomicroscopy

Long-term time-lapse microscopy analysis was performed on the culture under nonpermissive condition from day 0 to day 14 to follow the process of differentiation. Time-lapse microscopy was performed as previously described [18]. Briefly, two inverse microscopes, placed in a CO_2_ incubator, were equipped with high-sensitivity digital cameras and connected to a dual image-acquisition computer system. Illumination was developed to minimize heat- and phototoxicity. Operation of the spectrally cold-white light emitting diodes was synchronized with image-acquisition periods. Frames were recorded every minute and the whole video sequence was converted to database form. The time of exposure was indicated in the right lower corner of each frame. Exposures were converted to video films by speeding up the projection to 30 exposures/seconds.

### 2.4. Immunohistochemistry of Human Kidney Specimen

Formalin-fixed paraffin-embedded tissue in 4 *μ*m sections from the human kidney cortex was stained after antigen retrieval. The sections were subsequently incubated in 3% H_2_O_2_ for 15 minutes to quench endogenous peroxide activity and blocked for 20 minutes at room temperature to suppress nonspecific binding of subsequent reagents. Subsequent FtH visualization in podocytes in the glomeruli monoclonal antibody from Paolo Arosio (1 : 150; 6,5 *μ*g/mL) was used in combination with Wilms tumor 1 (WT-1) antibody (sc-192, 1 : 50; Sanra Cruz Biotechnology Inc., Heidelberg, Germany). The sections were incubated for 15 minutes with avidin-biotin-HRP complex before applying biotin labelled secondary antibodies. Vectastain Elite ABC dual detection system (PK-6102; Vector Laboratories Ltd., Peterborough, United Kingdom) was used following the manufacturer's guidelines. To visualize antigens of interest Vector VIP (purple: SK-4600; Vector Laboratories Ltd., Peterborough, United Kingdom) and DAB (diaminobenzidine, brown: SK-4100; Vector Laboratories Ltd., Peterborough, United Kingdom) were applied as substrates. Appropriate positive and negative control slides were analysed in parallel.

### 2.5. Giemsa Staining

Modified Giemsa staining was performed on parallel cell cultures at different time points. The monolayer of cells was fixed with methanol for 5 minutes. After air-drying, the cells were stained with freshly prepared Giemsa solution (Sigma-Aldrich) for 10 minutes. The redundant staining solution was washed away with distilled water and the samples were let to air-dry again. Pink colour represents cytoplasmic staining and blue nuclear staining. Magnification was 10x.

### 2.6. Isolation of Nuclei and Visualization of Chromatin Structures

To compare the cyclic character of chromatin unfolding and chromosome condensation of both nondifferentiated and differentiated podocytes, isolation of nuclei was performed as described previously [19]. Briefly, after reversal of cell permeabilization, the cells were treated with colcemid to prevent them from entering a new cell cycle. The trypsinized cells underwent osmotic swelling; then chromatin structures from the preparation of nuclei were spread over glass slides dropwise from a height of approximately 30 cm. Slides were air-dried and dehydrated using increasing concentrations of ethanol (70%, 90%, 95%, and 100%) and then mounted with DAPI containing antifade medium (EverBrite mounting medium, Biotium Inc., Hayward, CA, USA). Blue fluorescence of DAPI was monitored by fluorescence microscopy.

### 2.7. Activities of Antioxidant Enzymes

Nondifferentiated and differentiated podocytes were harvested in KH_2_PO_4_ buffer (100 mmol/L, pH 7.4). Cells were lysed by sonication on ice (3 × 5 s), and cell lysate was obtained by centrifugation (16.800 ×g, 10 min, 4°C). Enzyme activities were measured from cell lysates as described previously [20].

### 2.8. Detection of Human FtH by Western Blot

Confluent podocytes grown on 6-well plates originating from both permissive and nonpermissive conditions were treated for 1 hour with heme in HBSS (Hank's Balanced Salt Solution). After that, heme was removed and cells were incubated in RPMI supplemented with 10% FBS for further 8 hours. Cells were harvested as described previously [21] and equal amount of each sample (25 *μ*g protein/lane) was subjected to 6% nondenaturing PAGE. FtH was detected using human FtH monoclonal antibodies at a dilution of 1 : 1000 and horseradish peroxidase- (HRP-) conjugated goat anti-mouse IgG as secondary antibody at a dilution of 1 : 15000. Antigen-antibody complex was detected by a HRP chemiluminescence system according to the manufacturer's instructions (Amersham Biosciences Corp., Piscataway, NJ, USA). Quantification was performed using video densitometry (AlphaDigiDoc RT, Alpha Innotech Corp., San Leonardo, CA, USA).

### 2.9. Podocyte Cytotoxicity Assay

Nondifferentiated and differentiated cell cultures of human immortalized podocytes were grown on 96-well plates and exposed to heme (1.25–5 *μ*mol/L) or H_2_O_2_ (125–500 *μ*mol/L) or the combination of heme and H_2_O_2_ in HBSS. When used in combination, cells were pretreated with heme for 1 hour prior to H_2_O_2_ treatment. Appropriate vehicles were analysed in parallel. Cell viability was determined by MTT-reduction (3-[4,5-dimethylthiazol-2yl]-2,5-diphenyl-tetrazolium-bromide) measured at 590 nm.

### 2.10. TBARS Measurement

Nondifferentiated and differentiated human podocytes were grown on 6-well tissue culture plates. Cells were exposed to heme (1.25–5 *μ*mol/L) or H_2_O_2_ (125–500 *μ*mol/L) in HBSS for 4 hours. Cells were scraped in 200 *μ*L of KH_2_PO_4_ buffer (100 mmol/L, pH 7.4) containing SDS (6%). To each sample 400 *μ*L of TBARS reagent (0.375 g 2-thiobarbituric acid, 2.08 mL HCl, 15 mL 10% trichloroacetic acid, and distilled water to a final volume of 100 mL) was added. After incubating at 90°C for 30 minutes the samples were cooled down and extracted with 100 *μ*L n-butanol with vigorous vortexing. Phases were separated by centrifugation (2000 ×g, 20°C, 10 min), and optical density of the organic phase was measured at 532 nm.

### 2.11. Statistical Methods

All data were analysed using the GraphPad Prism 5 software (GraphPad Software Inc., San Diego, CA, USA). Differences between two groups were analysed by unpaired *t*-test. For multiple comparisons ANOVA method followed by* post hoc* Tukey's test was used. All results represent mean ± SEM of at least three independent experiments. *P* values <0.05 were considered statistically significant.

## 3. Results

### 3.1. Podocyte Growth and Differentiation under Nonpermissive (37°C) Condition

**Figure 1:**
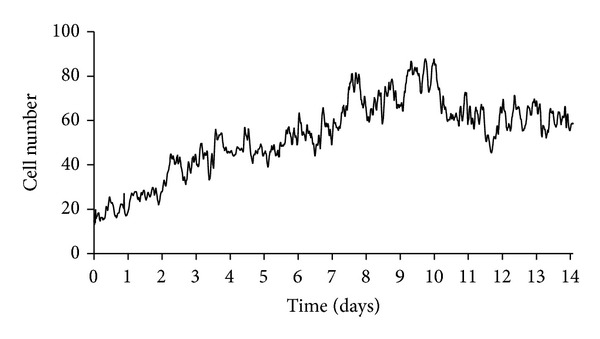
Podocyte growth under nonpermissive (37°C) condition. At 50–60% confluence, nondifferentiated cells were placed from 33°C to 37°C to induce differentiation which was followed by video microscopy for 14 days. Cell numbers were determined by using time-lapse phase contrast images that were acquired one frame every 10 minutes. Graph is a representative of 3 experiments with similar results.

**Figure 2:**
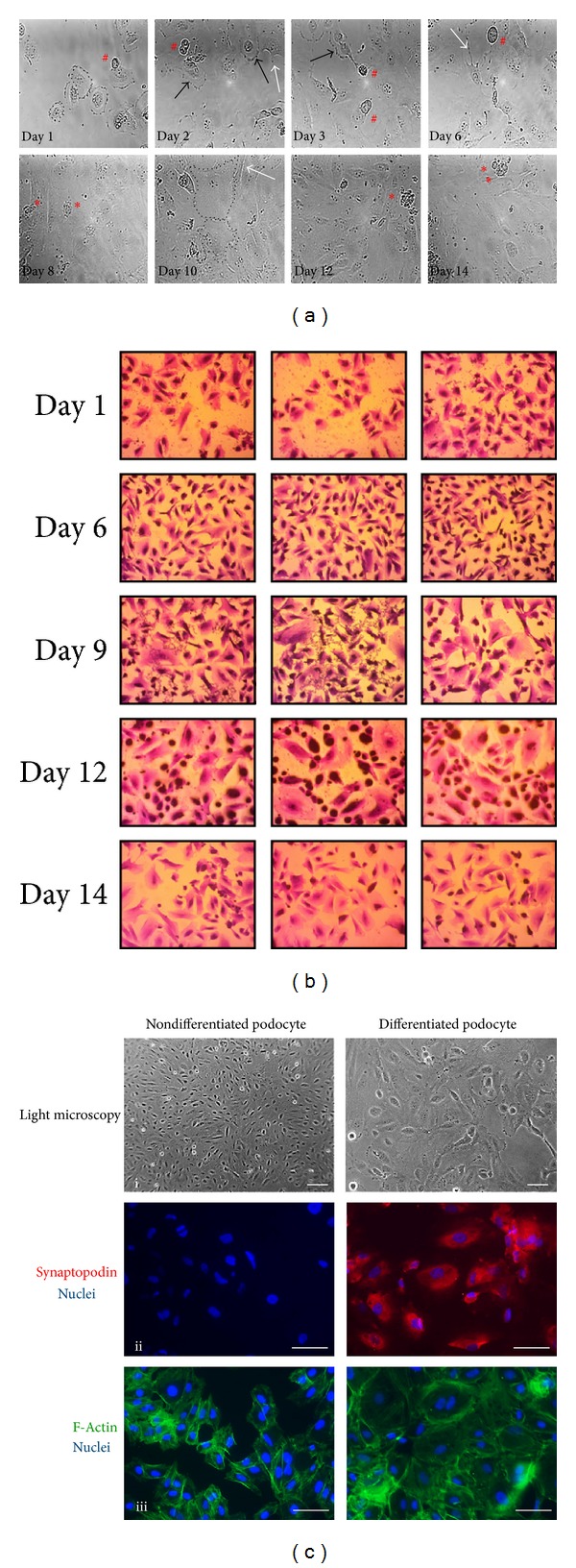
Podocyte differentiation under nonpermissive (37°C) condition. At 50–60% confluence, nondifferentiated cells were placed from 33°C to 37°C to induce differentiation which was followed by video microscopy for 14 days. (a) On selected time-lapse phase contrast images change in the shape and size of podocytes is indicated by broken outlines. Black arrows show shorter and rounded projections of differentiating podocytes. White arrows indicate longer and spindle like projections of arborizing podocytes. Mitotic cells, marked with red double sharps, become detached from the surface of the vessel for a while before the daughter cells adhere again to the bottom. Red asterisks denote apoptotic cells. (b) Modified Giemsa staining was performed on parallel cell cultures at different time points. Static visualization of the cell culture supports the findings of dynamic assessment by time-lapse monitoring. Cytoplasmic staining (pink), nuclear staining (blue). Magnification 10x. (c) Light microscopy and immunofluorescent microscopy images of cell cultures demonstrate characteristic features of human podocytes. Left panels represent nondifferentiated podocytes grown under permissive condition; right panels represent differentiated podocytes after 10 days of culturing under nonpermissive condition. (i) Light microscopy images, scale bars: 300 *μ*m, each. Nondifferentiated cells are small in size and show a cobble-stone like morphology, whilst differentiated cells have enlarged cell bodies with large nucleus, bulky cytoplasm, and ramifying processes. (ii) Synaptopodin-Cy3 (red) and DNA (DAPI, blue) are shown, scale bars: 300 *μ*m, each. Synaptopodin expression is present only in differentiated cells and shows filamentous staining. (iii) F-actin (phalloidin-FITC, green) and DNA (DAPI, blue) are shown, scale bars: 300 *μ*m, each. Images are representative of three experiments with similar results.

We placed nondifferentiated human podocytes expressing the thermosensitive A58 T antigen at 50–60% confluency from 33°C to 37°C to induce differentiation. We followed changes in cell number and morphology by video microscopy for 14 days. By analysing the growth curve we observed that the initial exponential growth phase lasted for 9 days (Figure 1). After cell number reached its peak between days 9 and 10, we saw a slight decrease in cell number followed by stabilization of the culture (days 10–14) when we could hardly detect any mitotic event. When the temperature was shifted to 37°C the morphology of nondifferentiated podocytes gradually transformed into that of differentiated cells (Figures 1 and 2). At permissive temperature (33°C) nondifferentiated podocytes showed typical epithelial cobblestone morphology. In contrast, differentiated podocytes are characterized by enlarged cell bodies with an irregular shape (Figure 2(a), broken outlines) and the formation of processes involving shorter and rounded (Figure 2(a), white arrows) as well as longer and spindle-like projections (Figure 2(a), black arrows). Formations of spindle-like projections were often preceded by partial retraction of cytoplasmatic protrusions. Many differentiated cells developed cellular hypertrophy, some of the cells died (Figure 2(a), red asterisks), and others started to arborize. We detected numerous binucleated but nonproliferating cells. After day 9 we registered a reduction of cell number, but the motility of cells remained high even after differentiation, and the culture proved capability of migration until all bare fields of the culturing vessel were completely covered.

To prove that differentiation of podocytes was complete during the 14 days, we evaluated synaptopodin expression in undifferentiated and differentiated cells by immunostaining (Figure 2(c), panel ii). Synaptopodin is a podocyte specific protein that plays an important role in the maintenance of cytoskeletal integrity [22], preserves the dynamic plasticity of foot processes, and thereby provides protection against proteinuria. We observed that synaptopodin expression was missing in nondifferentiated podocytes, while differentiated cells showed obvious expression of this molecule (Figure 2(c) ii panels, synaptopodin: red, nuclei: blue). The actin-based foot processes of kidney podocytes with the interposed slit diaphragms form the final barrier to proteinuria. We used the actin cytoskeleton marker phalloidin to visualize cytoskeletal rearrangement during differentiation (Figure 2(c) iii panels, actin: green, nuclei: blue).

### 3.2. Nuclear Expansion and Changes in Chromatin Structure during Podocyte Differentiation

**Figure 3:**
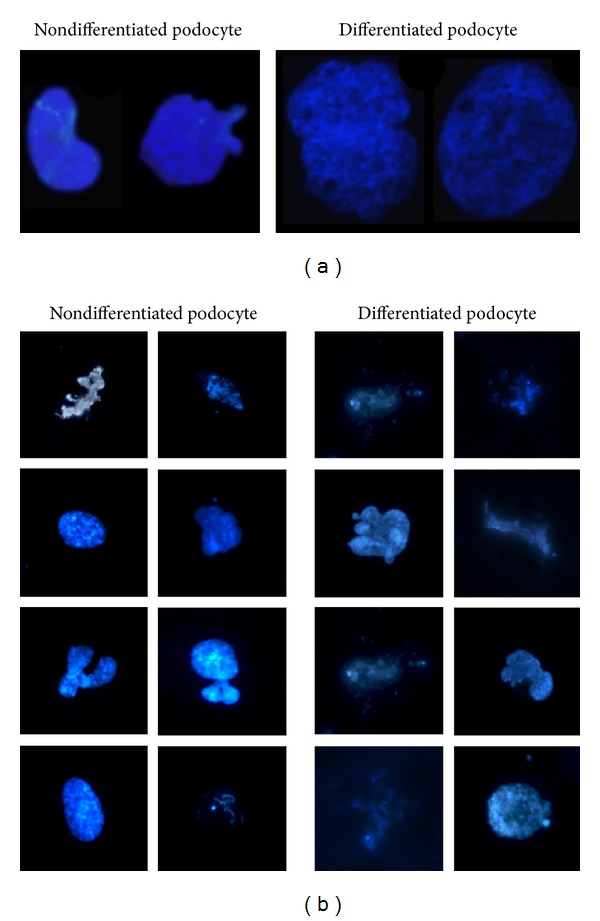
Nuclear expansion and changes in chromatin structure during podocyte differentiation. (a) Representative images show the nuclei of nondifferentiated human podocytes kept under 33°C (permissive condition, left) and the nuclei of differentiated podocytes grown for 14 days at 37°C (nonpermissive condition, right) stained with DAPI. The size of the nucleus increased during the process of differentiation. (b) Chromatin structures in immature and mature podocytes are apparently different. Nuclear preparation of undifferentiated cells contains visible chromosomes with more condensed chromatin structure and several foci of heterochromatin in contrast with the loosely packed chromatin from differentiated cells.

We observed significant increase both in cellular size and in nuclear size of differentiated podocytes relative to the cell body and nuclei of nondifferentiated cells.  Figure 3(a) shows small nondifferentiated (left) and large differentiated nuclei (right) of podocytes after staining with DAPI. This observation corresponds to the idea of a high transcription rate and a low or nonexistent replication rate of differentiated podocytes in the kidney. To further examine whether changes in nuclear size are associated with different chromatin structure, we stained chromatin with DAPI after lysing the nuclei of nondifferentiated and differentiated podocytes. In the nucleoplasm of undifferentiated podocytes there are several dispersed foci of heterochromatin where the transcription of genes is almost inactive (Figure 3(b)). Images also show visible chromosomes referring to a more condensed state of the chromatin, which indicates high mitotic activity. In comparison, in differentiated podocytes the organization of the chromatin structure is more homogeneous; fewer foci of heterochromatin are visible as actively transcribed genes are more loosely packaged. This finding testifies that a high rate of gene transcription and consequent protein synthesis is present in these cells. Tight chromatin packages and visible chromosomes can rarely be observed in mature cells, reflecting a weak tendency of differentiated podocytes to go through the cell cycle. Although it is known to be rare, mature podocytes under certain circumstances go through mitosis but they are unable to carry out cell division. This can be the explanation for the presence of bi- or multinucleated cells visualized by immunofluorescent dyes and very few condensed chromosomes in the nuclear preparations.

### 3.3. Mature Podocytes Are Highly Resistant to Oxidative Stress

**Figure 4:**
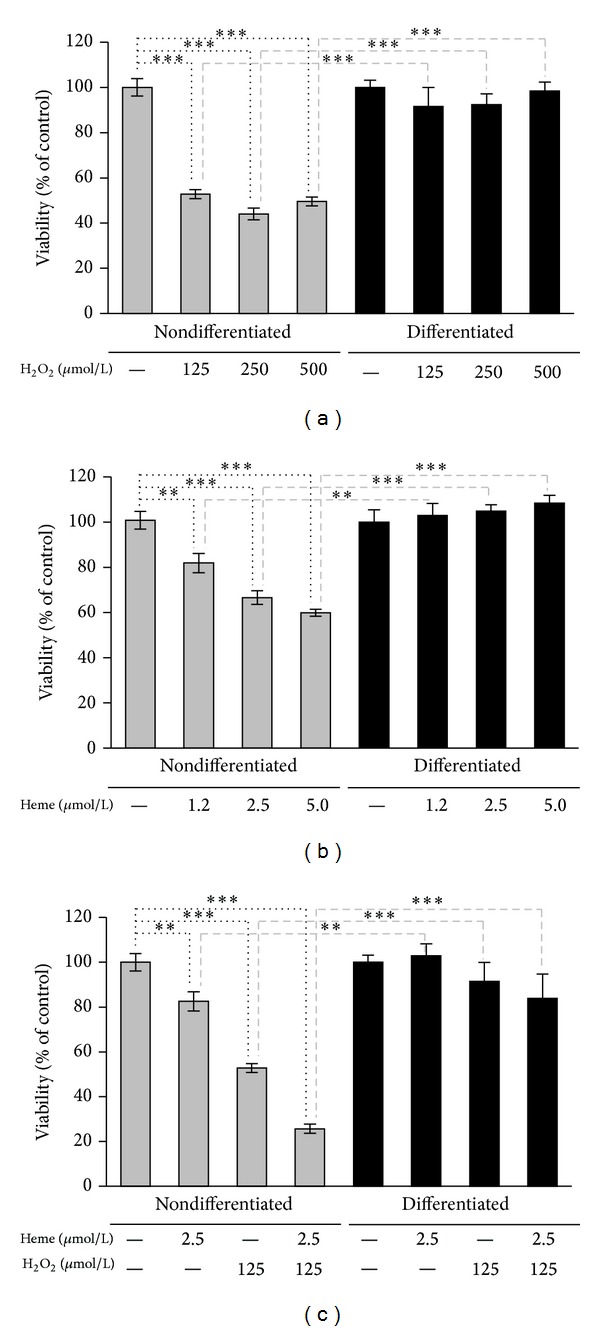
Mature podocytes are highly resistant to oxidative stress. (a) Nondifferentiated and differentiated podocytes were exposed to H_2_O_2_ (0–500 *μ*mol/L in HBSS) for 4 hours. Cell viability was assessed by MTT assay. (b) Nondifferentiated and differentiated podocytes were subjected to heme treatment (0–5 *μ*mol/L in HBSS). After 1 hour heme was replaced by HBSS. Cell viability was measured by MTT assay after a 4-hour incubation period. (c) Nondifferentiated and differentiated podocytes were treated with heme (2.5 *μ*mol/L in HBSS) or vehicle for 1 hour. After washing the heme away cells were exposed to H_2_O_2_ (125 *μ*mol/L in HBSS) or vehicle for 4 hours. Cell viability was determined by MTT assay. Results are shown as mean values ± SEM of at least three independent experiments each performed in quadruplicate. **P < 0.01, ***P < 0.001.

To compare oxidative resistance of undifferentiated and mature podocytes, we tested the effect of H_2_O_2_ on cell viability. Nondifferentiated cells were sensitive to H_2_O_2_ that caused about 50% of cell death at doses between 125 and 500 *μ*mol/L. In contrast, no cytotoxicity was observed in differentiated podocytes using the same doses of H_2_O_2_ (Figure 4(a)). Heme, as a prooxidant, can sensitize various cell types to oxidative stimuli, such as H_2_O_2_ [14] or inflammatory cytokines, such as TNF-alpha [23, 24]. We carried on checking the effect of heme alone or in combination with H_2_O_2_ on cell viability. As shown in Figure 4(b), heme alone caused death of nondifferentiated podocytes in a dose-dependent manner, while the same doses did not exert any cytotoxic effect to differentiated cells. Treatment of nondifferentiated podocytes with heme prior to H_2_O_2_ challenge led to decreased cell viability (53.3% versus 25.3%) (Figure 4(c)). In contrast, mature podocytes survived this lethal combination of heme and H_2_O_2_.

### 3.4. High Oxidative Resistance of Mature Podocytes Is Accompanied by Increased Activities of Antioxidant Enzymes

**Figure 5:**
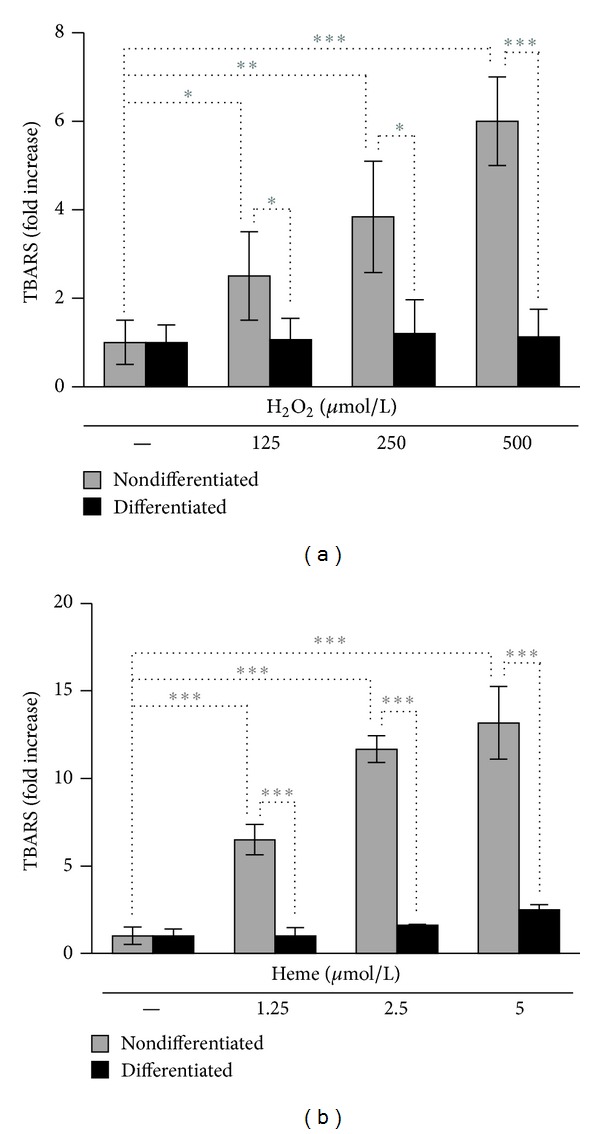
TBARS levels are elevated in nondifferentiated podocytes but not in differentiated cells under stress conditions. (a) Nondifferentiated and differentiated podocytes were exposed to H_2_O_2_ (0–500 *μ*mol/L in HBSS) for 4 hours, followed by measurement of TBARS levels. (b) Nondifferentiated and differentiated podocytes were subjected to heme treatment (0–5 *μ*mol/L in HBSS) for 4 hours, followed by measurement of TBARS levels. Results are expressed as fold increase over vehicle-treated controls and shown as mean values ± SEM of three independent experiments each performed in triplicates. *P < 0.05, **P < 0.01, and ***P < 0.001.

**Figure 6:**
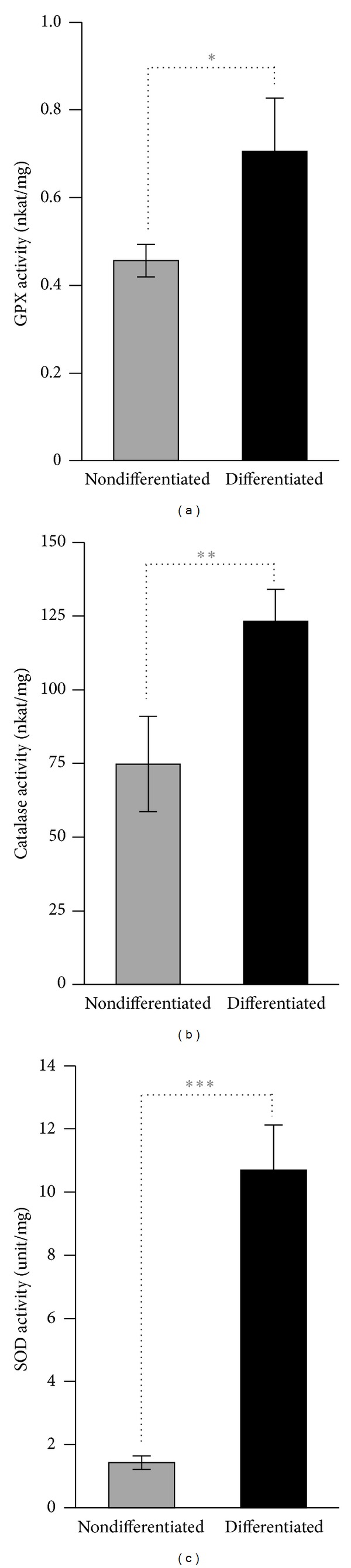
Antioxidant enzyme activities are elevated in mature podocytes. ((a)–(c)) GPX, catalase, and SOD activities of differentiated and nondifferentiated podocytes were measured. Results shown as mean values ± SEM of two independent experiments each performed in triplicates. *P < 0.05, **P < 0.01, and ***P < 0.001.

When challenged with H_2_O_2_ or heme nondifferentiated podocytes underwent cell death, whereas mature podocytes showed high resistance. To assess whether this phenomenon was accompanied by differences in oxidative stress, we measured the oxidative stress marker TBARS levels in both nondifferentiated and differentiated podocytes under normal and stress conditions. We found that nondifferentiated podocytes responded by a dose-dependent elevation of TBARS levels to both H_2_O_2_ and heme stress, whereas the same triggers did not cause significant increases in TBARS levels in differentiated podocytes (Figures 5(a) and 5(b)). Then we assessed whether the increased oxidative resistance of mature podocytes is associated with elevated activities of major antioxidant enzymes: GPX, catalase, and SOD. We found that activities of all the tested antioxidant enzymes were significantly higher in mature podocytes when compared to nondifferentiated cells (Figures 6(a)–6(c)).

### 3.5. FtH Expression Is High and Inducible in Differentiated Podocytes and Can Be Detected in Large Amount in Podocytes of Human Kidney Sections

**Figure 7:**
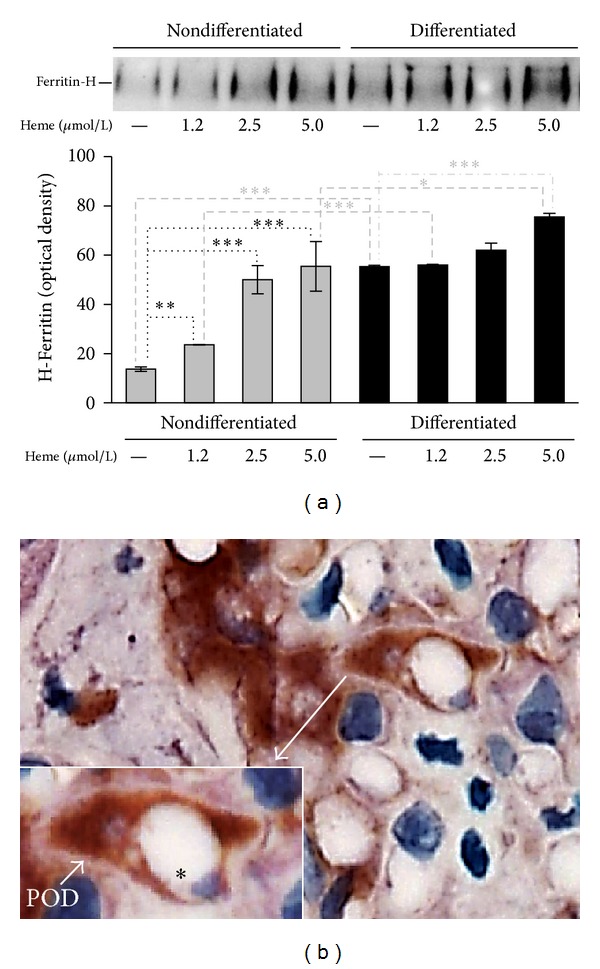
FtH is highly expressed in differentiated podocytes. (a) Nondifferentiated and differentiated podocytes were treated with heme (0–5 *μ*mol/L in HBSS) and harvested after 4 hours. Equal amounts of total protein (25 *μ*g) were applied to nonreducing gel for western blotting. Western blot showed that FtH is inducible and already present in both cell types at baseline; however, basic FtH expression is more robust in differentiated cells. Induction of FtH by heme treatment is more pronounced in nondifferentiated podocytes compared to differentiated cells. (b) FtH (brown) and WT-1 (purple) staining of native human kidney section are shown. Magnification demonstrates the lumen of a glomerular capillary (∗) and the cell body of a glomerular podocyte with its pedicles (POD) embracing the capillary wall in cross-section. Strong brown staining indicates the presence of FtH in the cytoplasm of podocytes. Scale bar: 10 *μ*m. Representative image of 3 with similar result.

To explore the underlying molecular mechanism of high oxidative resistance of differentiated podocytes, we compared the expression of FtH in nondifferentiated and differentiated podocytes in the absence or presence of heme. Because of its iron content, heme is a strong inducer of ferritin. First, by comparing basal FtH expressions, we found that differentiated podocytes express about 4 times more FtH than nondifferentiated cells (Figure 7(a)). Upon heme treatment, nondifferentiated podocytes upregulated FtH expression dose dependently up to a 4-fold increase at the highest dose of heme applied (5 *μ*mol/L). In contrast, in differentiated podocytes, which show high FtH expression at basal level, low doses of heme (1.2 and 2.5 *μ*mol/L) failed to induce FtH expression further more. Higher doses of heme triggered the induction of FtH suggesting that these differentiated podocytes remained responsive to this challenge but their resistance is shifted. Next, we examined whether FtH is expressed in significant amount in human kidney podocytes. Adult human kidney sections were stained with WT-1 antibody to visualize podocytes (purple) structurally in the glomerulus. For functional assessment, the sections were counterstained with FtH antibody. Glomeruli in the cortex contained several podocytes with strong FtH staining (brown), which supports our* in vitro* findings of high FtH content and iron sequestering capacity of resting podocytes (Figure 7(b)).

## 4. Discussion

The aim of this study was to explore different aspects of podocyte differentiation including changes in podocyte morphology, chromatin structure, cell motility, and oxidative stress resistance, as well as to find molecular explanation for the long life duration of podocytes in the kidney. Podocytes are highly specialized cells with an exquisite cell structure underlying their function [25]. During embryonal development of the human kidney, podocytes loose lateral attachments and form foot processes extending far from the cell bodies and resembling an interdigitated scaffolding around the glomerular capillaries [26]. The interdigitated foot processes in between with filtration slits are bridged by a slit diaphragm, which plays a major role in establishing the selective permeability of the glomerular filtration barrier [25]. Injury to podocytes leads to protein leakage, a common mechanism present in most glomerular diseases [27]. Most of our knowledge about podocyte differentiation comes from studies performed using fixed cells or cell extracts, which are usually used for molecular and biochemical assays. With the advances in time-lapse microscopy imaging, we can now study the dynamic interplay between living podocytes under special culturing conditions [18]. An immortalized podocyte cell line, which has been established and previously described [28], provided special opportunity to study those mechanisms, by which these cells survive in such a hectic environment almost for a lifetime [29]. Having undergone several mitotic events and a complete differentiation process, podocytes gained a different phenotype with huge cell bodies and longer lamellipodia [30]. The cytoskeletal architecture of the cells became more prominently visible even under light microscopy, with voluminous longitudinal bundles of actin filaments in the cell bodies and in the processes of the podocytes. The motility of cells remained high even after differentiation, and the culture proved capability of migration until full confluence of the culture was reached. A previous report comparing four cultured podocyte cell lines from different species also published consistent data on the high migration rate and motility of human podocytes [30]. As cultured podocytes progressively exited the cell cycle, parallelly they lost the ability to multiply themselves, that is, retrieve the function of damaged or dead podocytes. In conformity with the study by Liapis et al. [3], terminal differentiation implied permanent exit from the cell cycle; thus maturation process was accompanied by loss of mitotic activity and termination of podocyte replication. This may serve as explanation for our observation that the undifferentiated culture showed a higher proliferation rate than the differentiated one. Albeit podocyte progenitors (local parietal epithelial cells with the ability to replicate) support regeneration [31, 32], a great extent of cell detachment renders the maintenance of kidney function impossible, owing to the fact that mature podocytes have an infinite duration of life [18].

Formation of gradually lingering podocyte processes was also observed on time-lapse live-cell microscopy imaging. This finding is in agreement with the results of previous analyses, which determined that the vast majority of the long processes extending from cultured podocytes were generated by extension of lamellipodia followed by partial retraction leaving behind thin processes (spikes) [8].

To date, few studies have investigated and compared nuclear properties of glomerular podocytes. The primary role of the nucleus is to store information and ensure a site for cell retrieval and replication [33]. We studied the difference in the nuclei and the structure of chromatin between nondifferentiated and differentiated podocytes to assess their function in controlling gene expression and describe those characteristics that sustain the inability of differentiated cells to divide. Consistent with those described in the literature, we also found that the nuclear architecture, including the chromatin, is distinct in undifferentiated cells from differentiated cells in many respects. In conformity with previous observations [34], heterochromatin foci were larger and dispersed in undifferentiated cells. Under specific developmental signaling cues, this type of heterochromatin can loose its condensed structure and become transcriptionally active. There is a transient increase in number of these foci before cells enter terminal differentiation and exit the cell cycle, but their clustering takes place later [35]. Undifferentiated cells are characterized by hyperdynamic plasticity of chromatin proteins, fluid nuclei, physical nuclear plasticity, and global nuclear dynamics, supporting an open conformation model of chromatin in undifferentiated cells [34]. During the mitotic phase of the cell cycle, a partial condensation of the DNA occurs in prophase, so that the mitotic chromosomes occupy a much smaller volume of the nucleus. At telophase, the chromosomes swell again to fill the entire nucleus. We report that the nuclei of undifferentiated podocytes contain several foci of heterochromatin and also contain visible chromosomes beside the more condensed state of the chromatin, which refers to high mitotic activity. On the contrary, in differentiated podocytes the organization of the chromatin structure itself is more homogeneous; fewer foci of heterochromatin are visible as actively transcribed genes are more loosely packaged. This also supports the fact that there is a high gene expression rate in differentiated cells. We did not observe tight chromatin packages and visible chromosomes in mature cells reflecting a weak tendency of differentiated podocytes to go through the cell cycle.

Podocytes are subjected to several injurious and deleterious factors, for example, oxidative stress factors, while producing the glomerular filtrate. Oxidative stress resistance and the exact mechanisms by which these cells survive are yet uncovered. Here we report that the oxidative resistance of differentiated podocytes is much higher than that of undifferentiated cells against H_2_O_2_-induced oxidative stress. Because of the Fenton reaction, ROS-mediated oxidative stress is highly amplified in the presence of redox-active iron. Therefore, we tested oxidative resistance of differentiated and nondifferentiated podocytes in the presence of heme that act as a Fenton catalyst [36, 37]. When checking the effect of heme alone or in combination with H_2_O_2_ on cell viability, we found that there was a significant difference in the oxidative resistance capacity of undifferentiated and differentiated podocytes. Heme alone caused a strongly significant decrease in cell viability on nondifferentiated podocytes in a dose-dependent manner, while the same doses did not exert any cytotoxic effect on differentiated cells. Treatment of nondifferentiated podocytes with heme prior to H_2_O_2_ challenge resulted in an even lower rate of cell viability. In contrast, mature podocytes survived this lethal combination of heme and H_2_O_2_. H_2_O_2_ and heme-provoked cell death of nondifferentiated podocytes were accompanied by elevated TBARS levels, whereas the same triggers did not cause significant increases in TBARS levels in differentiated podocytes. Moreover, increased oxidative resistance of mature podocytes is associated with elevated activities of GPX, catalase, and SOD compared to nondifferentiated cells.

FtH has been shown to be effective in the defence against oxidative cell injury [7, 14]* via* sequestering iron from Fenton-like reactions. The deletion of FtH is embryonically lethal in mice; therefore, a conditional FtH deficient mice model was generated [16] to allow studying the role of FtH in different disease models. By using these mice, emerging* in vivo* evidence supports the idea that FtH provides protection to tissue damage. Recently, Zarjou et al. showed that conditional deletion of* FtH* in renal proximal tubules increased nephrotoxicity in different models of acute kidney injury [38]. Gozzelino et al. found that conditional deletion of* FtH* in the liver increases tissue damage and mortality in mice models of severe malaria [15]. Moreover, they showed that FtH expression is associated with reduced tissue damage in humans suffering from Plasmodium infection [15].

This finding is in agreement with our hypothesis that FtH may play a role in protecting podocytes from deleterious injuries. Differentiated podocytes that are more resistant to oxidative stress express more FtH than nondifferentiated cells. To assess the* in vivo* relevance of our observation, we examined the FtH content of human kidney specimens. Sections of kidney cortex rich in glomeruli were stained immunohistochemically. In support of our theory, we detected a massive FtH store in the cytoplasm of podocytes surrounding the glomerular capillaries that might be implicated in long-term survival of podocytes.

**Figure 8:**
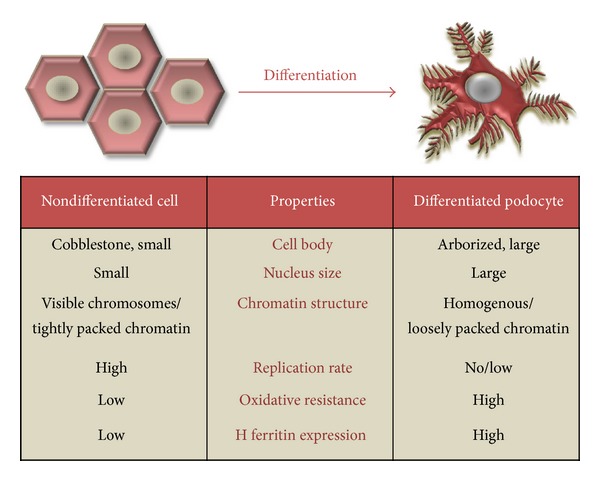
Schematic illustration of phenotype alterations during podocyte differentiation. Morphological changes characterized by alteration in cell body, nucleus size, and chromatin condensation accompanied by functional changes such as increase in oxidative resistance and FtH expression during podocyte differentiation are shown.

This study provides the first evidence that, during embryonic differentiation, podocytes go through fundamental morphological and functional changes assisting them to intensify defence mechanisms against oxidative stress damage and increase their resistance essential for long life duration (Figure 8). Antioxidant enzymes and FtH might contribute to the protection of human glomerular podocytes against oxidative injuries and highlight the so far unrevealed but certainly central importance of this protective system in glomerular diseases.

## Supplementary Material

Method: Time-Lapse Videomicroscopy. Long-term time-lapse microscopy analysis was performed on the culture under nonpermissive condition from day 0 to day 14 to follow the process of differentiation. An inverse microscope, placed in a CO_2_ incubator, was equipped with high-sensitivity digital cameras and connected to an image-acquisition computer system. Frames were recorded every minute and the whole video sequence was converted to database form. The time of exposure was indicated in the right lower corner of each frame. Exposures were converted to video films by speeding up the projection to 30 exposures/seconds.
Results: Podocyte Growth and Differentiation under Nonpermissive (37°C) Condition. We placed nondifferentiated human podocytes expressing the thermosensitive A58 T antigen at 50–60% confluency from 33°C to 37°C to induce differentiation. We followed changes in cell number and morphology by video microscopy for 14 days. we observed that the initial exponential growth phase lasted for 9 days. After cell number reached its peak between days 9 and 10,we saw a slight decrease in cell number followed by stabilization of the culture (days 10–14) when we could hardly detect any mitotic event.When the temperature was shifted to 37°C the morphology of nondifferentiated podocytes gradually transformed into that of differentiated cells. At permissive temperature (33°C) nondifferentiated podocytes showed typical epithelial cobblestone morphology. In contrast, differentiated podocytes are characterized by enlarged cell bodies with an irregular shape and the formation of processes involving shorter and rounded as well as longer and spindle-like projections. Formations of spindle-like projections were often preceded by partial retraction of cytoplasmatic protrusions. Many differentiated cells developed cellular hypertrophy, some of the cells died, and others started to arborize. We detected numerous binucleated but nonproliferating cells. After day 9 we observed a reduction of cell number, but themotility of cells remained high even after differentiation, and the culture proved capability of migration until all bare fields of the culturing vessel were completely covered.
